# Challenges in the Diagnosis of Multiple Sclerosis: A Case Report

**DOI:** 10.15388/Amed.2026.33.1.20

**Published:** 2026-07-22

**Authors:** Kristina Galinytė, Gintarė Butenytė, Gintarė Žemgulytė, Jurgita Makštienė, Renata Balnytė

**Affiliations:** 1 Lithuanian University of Health Sciences, Medical Academy, Faculty of Medicine, Kaunas, Lithuania; 2 Lithuanian University of Health Sciences, Medical Academy, Faculty of Medicine, Department of Neurology, Kaunas, Lithuania; 3 Lithuanian University of Health Sciences, Medical Academy, Faculty of Medicine, Department of Pathology, Kaunas, Lithuania

**Keywords:** tumor-like multiple sclerosis, differential diagnosis, CNS lymphoma, auglį primenanti išsėtinė sklerozė, diferencinė diagnostika, CNS limfoma

## Abstract

**Introduction:**

**:** Multiple sclerosis (MS) may present as a tumor-like, or tumefactive demyelinating lesion, that is indistinguishable clinically and radiologically from a brain tumor. This poses a diagnostic challenge.

**Case presentation:**

We present a case report of a 23-year-old woman who was hospitalized in the Neurosurgery Department for biopsy of a brain mass. She had been diagnosed with Hodgkin’s lymphoma at the age of 12, underwent surgery and chemotherapy, and had been in remission ever since. During her annual follow-up a magnetic resonance imaging (MRI) of the brain was performed that revealed a lesion in the right temporal lobe. It was interpreted as central nervous system (CNS) lymphoma. Before arranged neurosurgical intervention, a follow-up MRI scan revealed multiple new small lesions in both hemispheres. Biopsy was not performed, and the patient was referred to the Neurology Department for clarification of the diagnosis. No abnormalities were detected in the tests performed. Cerebrospinal fluid (CSF) was not tested due to the patient’s refusal to repeat the unsuccessful procedure. The patient was discharged from the hospital. Three months later, she arrived at the Emergency Department abroad with impaired coordination and hypoesthesia on the right side of the body. Two MRI scans revealed a new lesion in the left posterior periventricular area. CNS lymphoma was suspected again. The patient received treatment with levetiracetam and dexamethasone. Upon her return, an MRI scan was repeated, which revealed an enlargement of the lesion. CSF testing showed indistinct oligoclonal bands. As CNS lymphoma could not be excluded, a lesion biopsy was performed after discontinuation of the steroids therapy. Histological examination excluded malignancy. Later, an MRI scan was repeated; it showed two new contrast-enhancing lesions. Relapsing-remitting multiple sclerosis (RRMS) diagnosis was confirmed, and treatment with disease-modifying therapies (DMTs) was planned.

**Conclusions:**

Open-ring enhancement with little or no mass effect on MRI suggests a tumefactive demyelinating lesion (TDL). Moreover, TDLs tend to have a lower relative cerebral blood volume, and higher minimum and average apparent diffusion coefficient values compared to CNS lymphomas. Flow cytometry shows a monotypic B lymphoid population in cases of CNS lymphoma, not in MS.

## Abstract (it)

**Įvadas:**

Išsėtinė sklerozė gali pasireikšti į auglį panašiu demielinizuojančiu pažeidimu, kurį kliniškai ir radiologiškai sunku atskirti nuo centrinės nervų sistemos (CNS) limfomos. Tai lemia diagnostikos sunkumus.

**Atvejo pristatymas:**

Pristatome 23 metų moters, paguldytos į Neurochirurgijos skyrių smegenų audinio biopsijai, atvejo aprašymą. Hodžkino limfoma pacientei nustatyta, kai jai buvo 12 metų. Ji buvo operuota ir gydyta chemoterapija, ir nuo to laiko buvo remisija. Kasmetinio patikrinimo metu atliekant galvos magnetinio rezonanso tomografiją (MRT) dešiniojoje smilkininėje skiltyje aptiktas židinys. Jis interpretuotas kaip CNS limfoma. Prieš numatytą neurochirurginę intervenciją atliktas pakartotinis MRT tyrimas parodė daugybę naujų smulkių židinių abiejuose smegenų pusrutuliuose. Biopsija nebuvo atlikta, o pacientė nusiųsta į Neurologijos skyrių diagnozei patikslinti. Atlikus papildomus tyrimus, pokyčių nebuvo nustatyta. Smegenų skystis netirtas, pacientei atsisakius pakartoti anksčiau buvusią nesėkmingą procedūrą. Pacientė išleista gydytis ambulatoriškai. Po trijų mėnesių ji kreipėsi į kitos šalies skubios pagalbos skyrių dėl sutrikusios koordinacijos ir hipoestezijos dešinėje kūno pusėje. Atlikus du pakartotinius galvos MRT tyrimus, kairėje užpakalinėje periventrikulinėje srityje nustatytas naujas židinys. Vėl įtarta CNS limfoma, skirtas gydymas levetiracetamu ir deksametazonu. Pacientei grįžus, pakartotas galvos smegenų MRT tyrimas parodė židinio padidėjimą, o smegenų skystyje nustatytos neryškios oligokloninės juostos. Kadangi nebuvo galima atmesti CNS limfomos galimybės, nutraukus steroidų terapiją, atlikta židinio biopsija. Histologinis tyrimas paneigė piktybinį procesą. Vėliau atliktas pakartotinis galvos smegenų MRT tyrimas parodė du naujus, kontrastinę medžiagą kaupiančius židinius. Nustatyta recidyvuojančios remituojančios išsėtinės sklerozės diagnozė ir suplanuotas gydymas ligos eigą modifikuojančiais vaistais.

**Išvados:**

Atviras žiedinis kontrasto kaupimas ir nedidelis ar nenustatytas masės efektas MRT vaizduose būdingas į naviką panašiam demielinizuojančiam židiniui. Be to, šie židiniai pasižymi mažesniu santykiniu smegenų kraujo tūriu bei didesnėmis minimaliomis ir vidutinėmis tariamojo difuzijos koeficiento vertėmis, palyginti su CNS limfomomis. Tėkmės citometrija rodo monotipinę B limfoidinių ląstelių populiaciją esant CNS limfomai, bet ne išsėtinei sklerozei.

## Introduction

Multiple sclerosis (MS) is a chronic inflammatory and neurodegenerative disease of the central nervous system (CNS) which affects over 2.8 million people worldwide. It typically begins between the ages of 20 and 40, with women being more affected [1,2]. In most patients (85%), the disease starts as a relapsing-remitting course (RRMS), with the occurrence of relapses, and a potential progression to a secondary-progressive course over time. In the 15% remaining patients, MS evolves as primary-progressive course, which is characterized by steadily worsening symptoms, usually without relapses [3].

Diagnosing MS can be very challenging, as the disease may present with a wide range of neurological symptoms. Magnetic resonance imaging (MRI) has a key role in the diagnostic process, and it has been included as a fundamental paraclinical tool since the year 2017 revision of the McDonald diagnostic criteria [4]. This has substantially shortened the time to MS diagnosis and had led to earlier treatment with disease-modifying therapies (DMTs), limiting the accumulation of irreversible clinical disability [5]. Currently, the diagnosis of MS requires two conditions: a focal demyelinating pathology affecting at least two distinct CNS areas (dissemination in space [DIS]) occurring at separate times (dissemination in time [DIT]) [6]. However, McDonald criteria were not developed to distinguish MS from other conditions, but to identify it [7].

As a result, several diseases may resemble MS both clinically and on imagining [8]. These include infectious and cerebrovascular diseases, migraine, functional neurological disorders, spondylopathy, non-specific white matter lesions, neuromyelitis optica spectrum disorders, peripheral neuropathies, and neoplasm [9]. This is particularly challenging when a solitary demyelinating lesion greater than 2 cm is seen on neuroimaging. This lesion, defined as a tumefactive demyelinating lesion (TDL), often requires an invasive surgery and a brain mass biopsy to exclude malignancy [10,11]. When TDLs occur in the context of MS, the disease phenotype is called tumor-like or tumefactive multiple sclerosis (TMS) [11].

We present an uncommon case of a TDL as the inaugural manifestation of RRMS. In this case, making a definitive diagnosis was challenging given misleading clinical presentation, borderline oligoclonal bands (OCBs), inconclusive MRI scans, and a prior history of Hodgkin’s lymphoma. As CNS lymphoma could not be excluded, the stereotactic biopsy was performed as the last available diagnostic option. Only then was malignancy excluded. This case raises the question of how MS can be differentiated from CNS lymphoma, and whether biopsy remains the only option for the definitive diagnosis in such cases.

## Case presentation

**Figure 1:**
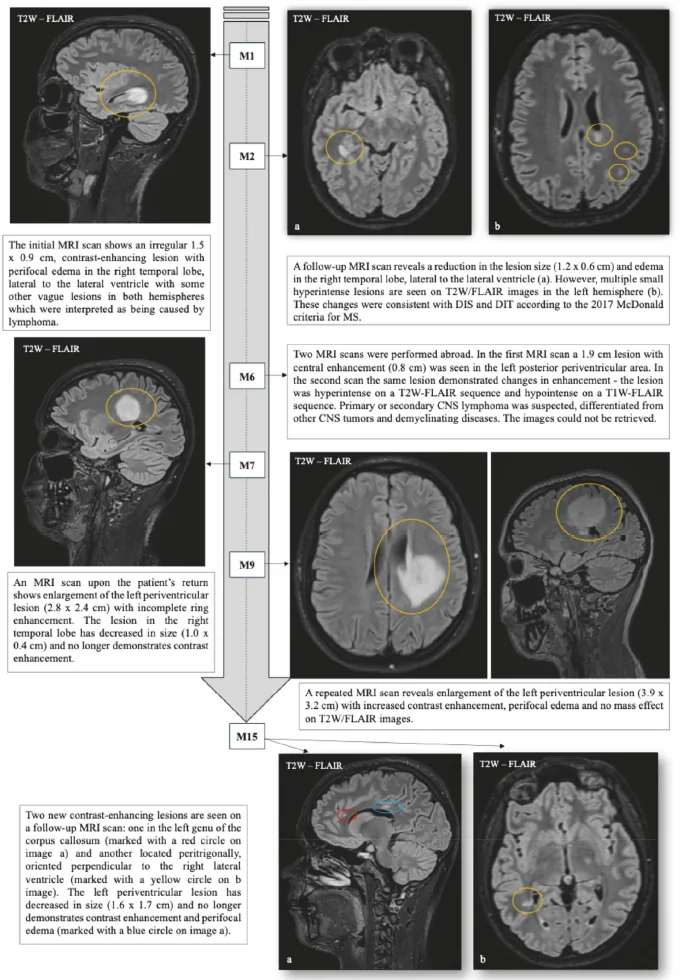
Graphical representation of magnetic resonance imaging changes from the onset of symptoms reported until the diagnosis. The letter ‘M’ followed by a number represents the month when the scan was performed. Magnetic resonance imaging sequences are specified in the upper left corner of each scan. Circles indicate the specific pathological feature discussed in the corresponding text below each scan *Note*. The following abbreviations are used: T2W-FLAIR, T2-weighted Fluid-attenuated inversion recovery; MRI, magnetic resonance imaging; M, month; cm, centimeter.

A 23-year-old woman was hospitalized in the Neurosurgery Department for biopsy of a brain mass. She had been diagnosed with Hodgkin’s lymphoma at the age of 12, underwent surgery and chemotherapy, and had been in remission ever since. During her annual follow-up, an MRI of the brain was performed due to complaints of dizziness. No other neurological symptoms were reported. The MRI scan revealed a 1.5 x 0.9 cm contrast-enhancing lesion with perifocal edema in the right temporal lobe with some other vague lesions in both hemispheres, which were interpreted as being caused by lymphoma (Figure 1, M1). A histological verification was required. One month later, before the arranged neurosurgical intervention, a follow-up MRI scan showed reduction of the lesion size (1.2 x 0.6 cm) in the right temporal lobe (Figure 1, M2) and multiple new small lesions in both hemispheres (Figure 1, M2) – the changes were consistent with DIS and DIT according to the year 2017 McDonald criteria for MS [6]. As a result of this radiological change, a demyelinating CNS disease was suspected, biopsy was not pursued, and the patient was referred to the Neurology Department. Neurological, neuro-ophthalmological, and otoneurological examination revealed no abnormalities. A lumbar puncture was performed with no cerebrospinal fluid (CSF) obtained, and the patient refused to repeat the procedure. Given the positive radiological change without the treatment, the patient was discharged from the hospital with plans for outpatient follow-up MRI scans and consultations.

Three months later, the patient arrived at the Emergency Department abroad due to impaired coordination and hypoesthesia on the right side of the body. Two MRI scans were performed during a two-week period. A 1.9 cm lesion was seen in the left posterior periventricular area, indicating possible primary or secondary CNS lymphoma differentiated from other demyelinating diseases and CNS tumors (Figure 1, M6). The patient received levetiracetam and dexamethasone, which she continued to use, and returned for a thorough examination. She was admitted to the Neurology Department. At that time, neurological examination revealed bilateral horizontal nystagmus, right lower limb ataxia and hemihypoesthesia of the right side. An MRI scan was repeated, revealing an enlargement of the left periventricular lesion (2.8 x 2.4 cm) with incomplete ring enhancement (Figure 1, M7). Further investigations were conducted. The blood test results showed positive immunoglobulin (Ig) G antibodies for cytomegalovirus (CMV) and Epstein-Barr virus (EBV), while antibodies against aquaporin (anti-AQP) and myelin oligodendrocyte glycoprotein (anti-MOG) were negative. Results of the lumbar puncture revealed normal white blood cell count, glucose, and protein levels, with indistinct OCBs, which were not detected in the blood serum. Anti-AQP, anti-MOG, and antibodies against Borrelia burgdorferi were not detected in the CSF.

**Figure 2:**
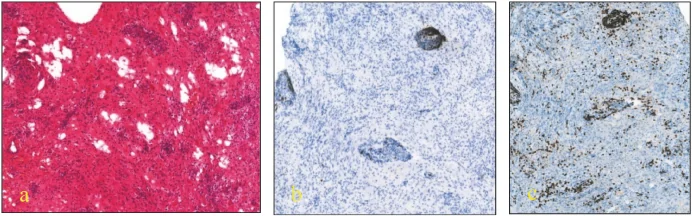
Histopathological examination of the brain tissue fragments stained with hematoxylin and eosin (a) reveals increased glial cellularity, perivascular infiltration with lymphocytes and macrophages. The walls of blood vessels walls are thickened, edematous, and, in some regions, infiltrated with lymphocytes. Myelin fibers appear thinned and, in some areas, degenerated. Immunohistochemistry of the brain tissue reveals expression of CD20+ (b) and CD3+ (c) cells

As CNS lymphoma could not be excluded, a follow-up MRI scan and a brain mass biopsy were planned after the discontinuation of a steroid therapy. A repeated MRI scan revealed an enlargement of the same lesion (3.9 x 3.2 cm), increased contrast enhancement and perifocal edema (Figure 1, M9). A stereotactic biopsy of the lesion was performed. Histopathological examination excluded malignancy and revealed morphological features consistent with an autoimmune or infectious process (Figure 2). The patient was then investigated on an outpatient basis for autoimmune encephalitis, paraneoplastic syndromes, and endocrine pathologies – and all test results were within normal ranges.

**Figure 3:**
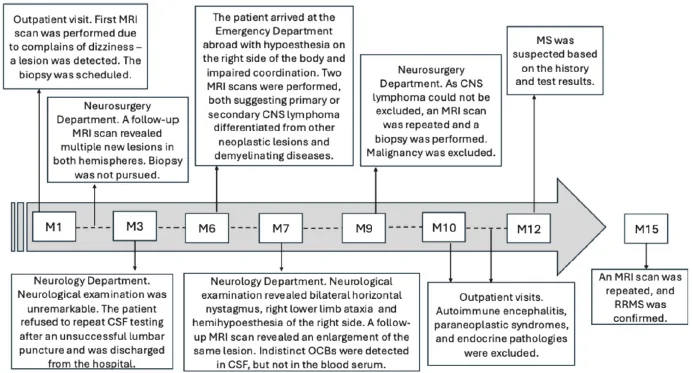
Timeline of the patient’s clinical history from the first symptoms to the confirmed diagnosis of relapsing-remitting multiple sclerosis *Note*. The following abbreviations are used: RRMS, relapsing-remitting multiple sclerosis; MRI, magnetic resonance imaging; CSF, cerebrospinal fluid; CNS, central nervous system; OCB, oligoclonal bands; MS, multiple sclerosis; M, month.

Based on the patient’s history and tests performed, MS was suspected. To fulfil the DIS and DIT criteria of McDonald 2017, an MRI scan was repeated three months later (Figure 1, M15). Two new contrast-enhancing lesions were identified, while the lesion where the biopsy was performed was found to have decreased (1.6 x 1.7 cm). According to the assessment of the medical history and the completed investigation, RRMS diagnosis was confirmed. Treatment with DMTs was planned. It took more than a year, 7 MRI scans, two lumbar punctures, multiple blood tests, and a brain mass biopsy with histological verification to confirm the diagnosis. A comprehensive description of the patient’s history in time is depicted in Figure 3.

## Discussion

TDL is a rare and distinct variant of the MS spectrum diseases that morphologically mimic primary brain tumors on MRI, posing a diagnostic challenge [12]. In this case, the initial lesion presented with imaging features consistent with a TDL, including a solitary mass of >2 cm, incomplete ring enhancement, perifocal edema, and absence of mass effect [13,14]. Nevertheless, CNS lymphoma was suspected due to the patient’s prior history of Hodgkin’s lymphoma. Unlike TDLs, CNS lymphomas tend to be larger and exert moderate-to-severe mass effect. They are also more likely to display complete ring enhancement, with either heterogenous or homogenous central enhancement, compared to TDLs [14,15]. Advanced imaging techniques can further assist in the differentiation. The dynamic susceptibility contrast (DSC) technique is the most used to distinguish CNS lymphoma from non-neoplastic lesions by using the relative cerebral blood volume (rCBV) as an indicator of neovascularization. TDLs tend to have a lower rCBV, but a high rCBV does not rule it out [16]. Diffusion tensor imaging (DTI) can also improve diagnostic sensitivity, as TDLs show higher minimum and average apparent diffusion coefficient (ADC) values compared to CNS lymphomas [17]. Combined with typical MRI features of TDLs, the findings based on these advanced imaging techniques increase the probability and diagnostic confidence that a lesion is actually a TDL.

While the above-mentioned features could assist in distinguishing the diseases, the clinical presentation remains non-contributory. A retrospective study of 133 patients with either TMS or CNS lymphoma supports this notion [18]. Another retrospective study of 87 cases found hemiparesis or hemiplegia (67%), sensory disorders (38%), dizziness (26%), and optic neuritis (24%) to be the most common TMS symptoms. Only one case of a ‘silent’ TMS has been reported, with a mild tremor of the hands as the only symptom [14]. This case represents another example of the dissociation between radiological and clinical presentation, as the patient initially complained about dizziness despite a large lesion in the right temporal lobe. Later, when a new a large lesion in the left periventricular area was detected, the patient’s neurological examination revealed bilateral horizontal nystagmus, right lower limb ataxia and hemihypoesthesia of the right side. This further complicated the differential diagnosis. Another complicating factor in this case was the patient’s response to corticosteroid therapy. Steroids usually tend to decrease the lesion size in 45–80% of TMS cases and 40–80% of CNS lymphoma cases [12,19]. This effect was not observed in the presented case, since the lesion size increased from 1.9 cm to 2.8 cm during one month of steroid therapy.

Another useful and essential adjunct in distinguishing a demyelinating disease from CNS lymphoma is CSF analysis. In this case, it showed a normal white blood cell count, glucose, and protein levels, with indistinct OCBs that were not detected in the serum. The presence of unmatched OCBs (OCBs found in CSF, but not in the blood serum) may reassure in favor of demyelination, and this finding may substitute for the traditional DIT criterion [20,21]. In addition to OCBs, an elevated immunoglobulin G (IgG) index has served as a marker for intrathecal IgG synthesis [22]. However, the year 2024 revised McDonald criteria now include the kappa free light chain (kFLC) index as an alternative to OCBs [23]. Nevertheless, OCBs are not specific to demyelination and can be found in other inflammatory or infectious conditions, as well as in cases of CNS lymphoma [21,24]. For the differentiation between a demyelinating disease and CNS lymphoma, evaluation of CSF cytology and flow cytometry could be beneficial. In cases of CNS lymphoma, CSF cytology reveals large lymphoid cells with irregular nuclei, basophilic cytoplasm, and prominent nucleoli, while the flow cytometry identifies a monotypic B lymphoid population with light chain restriction [25]. These changes are not observed in cases of a demyelinating disease. CSF biomarkers could assist, too. Median CSF interleukin (IL)-10 and soluble IL-2 receptor (sIL-2R) levels were found to be higher in CNS lymphoma patients compared to patients with a demyelinating disease, whose levels were within the normal range [12,26]. These markers, together with CSF cytology and flow cytometry, may aid in the differential diagnosis of these conditions.

All the above-mentioned methods only increase the likelihood that a lesion is demyelinating rather than malignant. None can fully exclude a CNS lymphoma, which is why biopsy, despite being invasive, remains the gold standard. However, misdiagnosis can occur even with biopsy due to the site of sampling, the amount of material taken, and misinterpretation of the sample in up to 31% of cases [27,28]. In our case, biopsy was performed as the last available option, given the unremarkable clinical presentation, inconclusive MRI findings, borderline OCBs, paradoxical response of the lesion to the steroids, and a history of Hodgkin’s lymphoma. One study of case series showed that 6 out of 7 TMS patients required a biopsy for the definitive diagnosis [11]. The histological findings in our case were increased glial cellularity, perivascular infiltration with lymphocytes (mainly CD3+ T cells, but CD20+ B cells were seen as well) and macrophages, thickened, edematous blood vessel walls, thinned myelin fibers and, in some areas, degenerated myelin fibers. Similar histological appearance has been reported in other TDL cases [11,12,29]. Although histological examination excluded malignancy, for the definitive diagnosis of a demyelinating disease, other infectious and autoimmune diseases were excluded as well.

It remains unclear whether past treatment of Hodgkin’s lymphoma could have contributed to the development of MS. It has been suggested that both diseases share epidemiologic characteristics, and only the young-adult-onset Hodgkin’s lymphoma (ages 15–39 years) has been associated with MS [30,31]. Shared association may be manifested due to genetic factors, such as the HLA DR2 allele, and a shared infectious etiology, particularly EBV [31]. Our patient had positive IgG antibodies against EBV. However, this result is not unexpected, since 90% of people are infected with EBV worldwide [32]. Moreover, it is unclear when the patient was infected with EBV, and whether the virus played any role in the development of either pathology.

This case demonstrates the transition from an isolated TDL to a RRMS [23]. The initial presentation was a solitary TDL, but the subsequent development of periventricular and juxtacortical lesions over time fulfilled the DIS and DIT criteria [23]. To confirm the diagnosis, it was necessary to exclude CNS lymphoma, a major clinical concern, that was ultimately ruled out via a brain mass biopsy. Such an invasive method should be considered when MRI findings are atypical (e.g., significant mass effect or complete ring enhancement), CSF analysis is negative for OCBs, there is a lack of response to steroid therapy, or there is a high suspicion of CNS malignancy [33]. When typical TDL features are seen on MRI, it is suggested to evaluate rCBV and ADC values along with CSF IL-10, sIL-2R levels before deciding on a biopsy [12,26]. In the presence of borderline OCBs, which are defined as CSF-restricted bands that do not meet the formal diagnostic criteria for positivity (≥2 bands) [34], testing the kFLC index is recommended in order to satisfy the CSF criterion [23].

## Conclusions

The differentiation between a TDL and CNS lymphoma remains a significant diagnostic challenge. On MRI, TDLs display incomplete ring enhancement with little or no mass effect. When MRI features are inconclusive, testing rCBV, minimum and average ADC values is beneficial. TDLs tend to have a lower rCBV, and higher minimum and average ADC values compared to CNS lymphomas. Evaluating CSF cytology and flow cytometry can also be helpful, as a monotypic B lymphoid population is associated with CSF lymphoma rather than demyelination. Testing CSF biomarkers could be beneficial, too, as median values of IL-10 and sIL-2R are higher in CNS lymphoma cases. Although these methods can contribute to the differential diagnosis, if the diagnosis remains inconclusive, a biopsy should be performed. It should be noted that this is a single illustrative case, and the proposed diagnostic approach should guide, but not replace, individualized decision-making.
